# Recent Advances on the Role and Therapeutic Potential of Regulatory T Cells in Atherosclerosis

**DOI:** 10.3390/jcm10245907

**Published:** 2021-12-16

**Authors:** Toru Tanaka, Naoto Sasaki, Yoshiyuki Rikitake

**Affiliations:** 1 Laboratory of Medical Pharmaceutics, Kobe Pharmaceutical University, Kobe 658-8558, Japan; t-tanaka@kobepharma-u.ac.jp (T.T.); rikitake@kobepharma-u.ac.jp (Y.R.); 2 Division of Cardiovascular Medicine, Department of Internal Medicine, Kobe University Graduate School of Medicine, Kobe 658-8558, Japan

**Keywords:** atherosclerosis, immunology, inflammation, regulatory T cells

## Abstract

Atherosclerotic diseases, including ischemic heart disease and stroke, are a main cause of mortality worldwide. Chronic vascular inflammation via immune dysregulation is critically involved in the pathogenesis of atherosclerosis. Accumulating evidence suggests that regulatory T cells (Tregs), responsible for maintaining immunological tolerance and suppressing excessive immune responses, play an important role in preventing the development and progression of atherosclerosis through the regulation of pathogenic immunoinflammatory responses. Several strategies to prevent and treat atherosclerosis through the promotion of regulatory immune responses have been developed, and could be clinically applied for the treatment of atherosclerotic cardiovascular disease. In this review, we summarize recent advances in our understanding of the protective role of Tregs in atherosclerosis and discuss attractive approaches to treat atherosclerotic disease by augmenting regulatory immune responses.

## 1. Introduction

Atherosclerosis provokes serious cardiovascular diseases (CVDs), such as ischemic heart disease and stroke, that are prominent causes of mortality worldwide. Although patients at high risk of atherosclerotic CVD usually receive state-of-the-art intensive treatment, there remains much residual risk of this disease. Experimental and clinical studies have provided firm evidence that innate and adaptive immune responses are critical factors for provoking vascular inflammation and atherosclerosis [1,2]. Although accumulating evidence suggests that vascular inflammatory responses could be responsible for the residual risk of atherosclerotic disease, we have no clinical therapies aimed to directly suppress inflammatory reactions in atherosclerotic lesions [3].

The first clinical evidence to prove the involvement of inflammation in atherosclerotic disease was provided by the Canakinumab Anti-inflammatory Thrombosis Outcomes Study (CANTOS) trial, clearly showing that treatment with a fully human monoclonal antibody targeting interleukin (IL)-1β reduced the cardiovascular disease risk in patients with old myocardial infarction (MI) [4]. Other evidence has been provided by recent clinical trials, the Colchicine Cardiovascular Outcomes Trial (COLCOT) [5] and low-dose colchicine (LoDoCo2) [6], demonstrating that treatment with colchicine, an anti-inflammatory drug indicated for the treatment of gout, familial Mediterranean fever, and pericarditis, resulted in a significantly lower risk of cardiovascular events in patients with recent MI or chronic coronary disease. These clinical trials highlight the efficacy of anti-inflammatory therapies as potentially feasible strategies to treat CVD at least in patients with past disease history.

Experimental and clinical data suggest that in addition to innate immune responses, adaptive immune responses, particularly T cell-mediated immune responses, have detrimental or protective roles in atherosclerosis depending on the cell types or experimental conditions [7]. Firm evidence indicates that regulatory T cells (Tregs), responsible for maintaining immunological tolerance and suppressing excessive immune responses [8], play an important role in preventing the development and progression of atherosclerotic disease through the regulation of pathogenic effector T cell (Teff) immune responses [9]. Several strategies to prevent and treat atherosclerosis via promoting regulatory immune responses have been developed [10]. Tuning the balance between proatherogenic Teffs and atheroprotective Tregs could be an effective therapeutic strategy for atherosclerotic disease.

In this review, we summarize recent advances in our understanding of the protective role of Tregs in atherosclerosis, and discuss attractive approaches to treat atherosclerotic disease by augmenting regulatory immune responses.

## 2. Role of the Immune System in Atherosclerotic Disease

**Figure 1:**
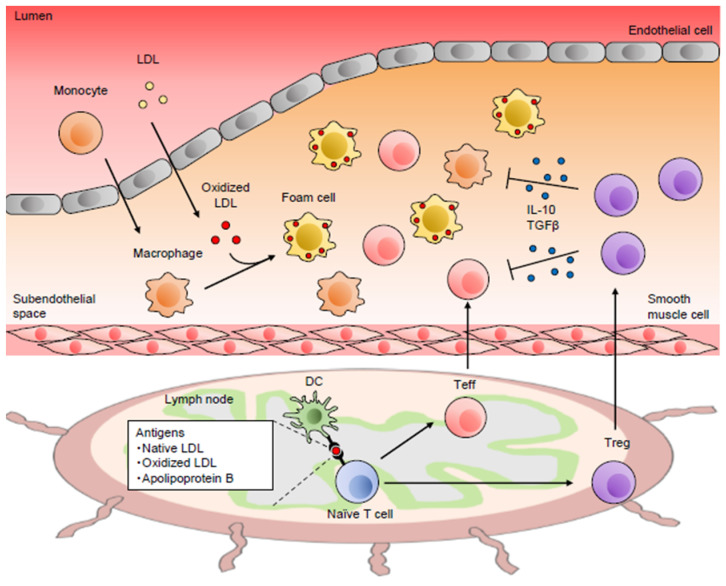
Role of the immune system in atherosclerosis. Chronic vascular inflammation via immune dysregulation is critically involved in the pathogenesis of atherogenesis. Regulatory T cells (Tregs) protect against atherosclerosis by suppressing activation of various immune cells. DC, dendritic cell; IL, interleukin; LDL, low-density lipoprotein; Teff, effector T cell; TGF, transforming growth factor.

Immune dysregulation causes chronic inflammation in the arterial wall, which is a key feature of atherosclerosis [11] (Figure 1). It has been suggested that the dysregulated arterial immunoinflammatory responses could induce atherosclerotic plaque instability and eventually lead to severe clinical events, including acute coronary syndrome (ACS) and stroke. It is believed that as the initial inflammatory response in atherosclerosis, accumulation and retention of low-density lipoprotein (LDL) in the arterial intima could occur. Upon endothelial activation, inflammatory monocytes enter the subendothelial space or the intima of the arterial wall, differentiate into macrophages, and form lipid-laden foam cells via uptake of modified LDL particles, leading to the promotion of arterial immunoinflammatory responses including proatherogenic Teff immune responses. A large number of experimental and clinical studies have revealed that various immune cells that mediate innate and adaptive immunity are deeply involved in the pathogenesis of atherosclerosis [1]. In particular, genetically altered mouse models of atherosclerosis including the apolipoprotein E-deficient (*Apoe*^−/−^) [12] and low-density lipoprotein receptor-deficient (*Ldlr*^−/−^) mice [13] have substantially contributed to the development of the atherosclerosis research field.

Naïve T cells are presented with some atherosclerosis-associated antigens by antigen-presenting cells (APCs), and differentiate into activated Teffs, including CD4^+^ or CD8^+^ T cells that accumulate into atherosclerotic lesions of atherosclerosis-prone *Apoe*^−/−^ or *Ldlr*^−/−^ mice and humans. Dendritic cells (DCs) have a capacity to present antigens and play complex roles in atherosclerosis. Various subsets of DCs have been shown to play protective or detrimental roles in atherosclerosis, depending on their subsets [14]. The finding of oligoclonal expansion of T cells in mouse atherosclerotic lesions indicates that T cells may expand in an antigen-specific manner within atherosclerotic plaques [15]. Several self-antigens, native LDL, oxidized LDL, and the major component of LDL, such as apolipoprotein B (ApoB), could provoke autoimmune responses in atherosclerotic lesions and be involved in the pathogenesis of atherosclerosis [16]. However, the role of these atherosclerosis-specific antigens in the development of atherosclerosis remains obscure, and a detailed analysis is needed. With the help of T cells and antigenic stimulation, B cell differentiation and activation occur. Recent experimental studies have revealed that several subsets of B cells play differential and complex roles in atherosclerosis [17]. Conventional B2 cells contribute to proatherogenic immune responses, while B1 cells or regulatory B cells protect against atherosclerosis by secreting natural IgM antibodies against oxidation-specific epitopes or IL-10, respectively.

Depending on the expression of specific transcriptional factors or variations in the local cytokine milieu along with antigen presentation, naïve CD4^+^ T cells differentiate into different Teff lineages, including T helper type 1 (Th1), T helper type 2 (Th2), and T helper type 17 (Th17) lineages, which have critical roles in the development of atherosclerosis in mice and humans [1]. Recent experimental and clinical studies using sophisticated methodologies, such as single-cell RNA sequencing and mass cytometry revealed that a distinct subset of T cells is the major cellular component in atherosclerotic plaques of mice [18] and humans [19]. Th1 cells specifically express the transcription factor T-box expressed in T cells (T-bet) and secrete pro-inflammatory cytokines, interferon (IFN)-γ, IL-2, and tumor necrosis factor (TNF)-α. Among these cytokines, the major Th1 cytokine, IFN-γ, has been shown to have potent pro-atherogenic effects [20,21]. Th1 cells predominantly exist in mouse [22] and human [23] atherosclerotic plaques and promote atherosclerosis. Th2 cells specifically expressing the transcription factor GATA3 secrete IL-4, IL-5, IL-10, and IL-13, and its major cytokine is IL-4. The genetic deletion of IL-4 in *Ldlr*^−/−^ mice reduces atherosclerosis [24], while exogenous administration of IL-4 did not affect atherosclerosis in *Apoe*^−/−^ mice [25], producing discrepant results. The role of Th2-mediated immune responses in atherosclerosis is heavily influenced by the secreted specific cytokines or animal models and remains controversial. Th17 cells specifically expressing the transcription factor RAR-related orphan receptor (ROR)-γt produce pro-inflammatory cytokine IL-17, and have been shown to substantially contribute to the development of several autoimmune diseases [26]. However, pharmacological inhibition of IL-17 function or its genetic inactivation yielded discrepant outcomes in atherosclerosis [27,28,29]. The role of Th17 cells in atherosclerosis may vary depending on animal models or experimental conditions, and further investigations are required. CD8^+^ T cells also infiltrate into atherosclerotic lesions, and their numbers are higher than CD4^+^ T cells in advanced human atherosclerotic lesions [19]. Experiments performing CD8α or CD8β monoclonal antibody-mediated depletion of CD8^+^ T cells in *Apoe*^−/−^ mice or their transfer into lymphocyte-deficient *Apoe*^−/−^ mice revealed that CD8^+^ T cells promote the development of atherosclerotic lesions with an unstable plaque phenotype [30]. On the other hand, depletion of CD8^+^ T cells by injecting CD8α-depleting antibodies in *Ldlr*^−/−^ mice resulted in less stable plaques characterized by reduced collagen content and increased macrophage content and necrosis, though that did not affect atherosclerotic lesion size [31]. Thus, CD8^+^ T cells have pro- and anti-atherogenic actions depending on experimental models.

T cell activation occurs after receiving two signals from APCs that involve interaction of the T cell receptor (TCR) with antigenic peptide/major histocompatibility complex (MHC) ligand and costimulation. The costimulatory signals promote or inhibit the TCR-MHC signal, depending on the type of costimulation. The T cell costimulatory and coinhibitory pathways play crucial roles in modulating functions of Teffs and Tregs and their balance, and these pathways have been shown to be involved in the pathogenesis of atherosclerosis in recent animal studies using genetically modified mice or neutralizing antibodies [32]. Activated T cells and forkhead box P3 (Foxp3)^+^ Tregs highly express the coinhibitory molecule cytotoxic T-lymphocyte-associated antigen-4 (CTLA-4) that binds to B7 family molecules, B7-1 (CD80) and B7-2 (CD86), on APCs and interrupts the interaction of these molecules with CD28 on T cells, resulting in potent suppression of T cell activation [33]. Our recent studies using CTLA-4 transgenic mice on an *Apoe*^−/−^ background in which T cells constitutively express CTLA-4 demonstrated that CTLA-4 overexpression protected against the development of atherosclerosis [34], abdominal aortic aneurysm (AAA) [35], and kidney disease [36] by suppressing maturation of DCs and pro-inflammatory Teff immune responses. CTLA-4-Ig that binds to CD80 and CD86 and has an inhibitory effect on the CD80/CD86–CD28 costimulation pathway is clinically effective in treating rheumatoid arthritis [37], and has also been shown to protect against experimental atherosclerosis [38]. These reports suggest that CTLA-4 could be a possible therapeutic target for atherosclerosis. Another important coinhibitory pathway is the interactions between programmed cell death protein 1 (PD-1) and programmed cell death ligand 1 (PD-L1)/PD-L2. Genetic deletion of both PD-L1 and PD-L2 [39] or of their receptor PD-1 [40] in *Ldlr*^−/−^ mice led to accelerated development of atherosclerotic lesions with increased accumulation of intraplaque CD4^+^ and CD8^+^ T cells, indicating an atheroprotective role of the PD-1–PD-L1/PD-L2 pathway.

In recent years, the new field termed immuno-oncology has emerged, and immunotherapies for various types of cancer have attained great success. Monoclonal antibodies to block PD-1, PD-L1, or CTLA-4, called immune checkpoint inhibitors (ICIs), have been developed. Recent experimental and clinical evidence suggests that stimulation of the antitumor functions of T cells with these ICIs is quite effective for the treatment of cancer, and has become a major treatment for advanced unresectable cancer [41]. In consideration of findings obtained from animal studies showing that these inhibitory molecules are negative regulators of atherosclerosis [34,38,39,40], treatment with ICIs have the potential to accelerate atherosclerosis in cancer patients who could have a higher risk of CVD. Importantly, a recent clinical trial has demonstrated that treatment with ICIs was associated with a higher risk of atherosclerosis-related cardiovascular events in patients with various types of cancer, providing evidence that T cell activation is critically involved in the development of atherosclerotic disease in humans [42]. Careful management is required for the use of ICIs for treating cancer patients with cardiovascular risk factors.

## 3. Protective Role of Tregs in Atherosclerotic Disease

Thymus-derived natural Tregs were discovered by Sakaguchi et al. in 1995 [43]. Tregs constitutively express high levels of CD25 (IL-2 receptor α-chain) molecule [43] and the transcription factor Foxp3, an essential factor for their differentiation and function [44,45]. Impaired Treg function has been shown to be a primary cause of several autoimmune diseases in mice [46] and humans [47], indicating that this cell population plays an indispensable role in the dominant suppression of autoimmune responses and the maintenance of immune homeostasis. Tregs have multiple inhibitory actions, including suppression of autoimmune T cell proliferation and its differentiation into Th1, Th2, and Th17 lineage, and the inactivation of various immune cells, including B cells, DCs, and macrophages [48]. Several lines of evidence indicate that Tregs protect against atherosclerosis by regulating pathogenic immunoinflammatory responses (Figure 1 and Table 1). Foxp3^+^ Tregs could also be generated from naïve T cells in the periphery, such as gut-associated lymphoid tissue (GALT), which are called peripherally derived Tregs (pTregs) that maintain mucosal immune tolerance and suppress autoimmune responses [49]. However, the differences in their characteristics between thymus-derived natural Tregs and pTregs remain obscure, and further investigation will be needed. For more detailed information about the diverse role of Tregs in immunology, the reader is referred to a recent review [8].

### 3.1. Protective Role of Tregs in Experimental Atherosclerosis

The costimulatory pathway CD80/CD86–CD28 plays a role in the generation and homeostasis of Tregs [50]. Genetic deficiency of this costimulatory signaling exacerbated atherosclerosis in *Ldlr*^−/−^ or *Apoe*^−/−^ mice, and was associated with a significant decrease in CD4^+^CD25^+^ Treg numbers in lymphoid tissues [51]. Notably, adoptive transfer of CD4^+^CD25^+^ Tregs abrogated the detrimental effects on atherosclerosis observed in *Apoe*^−/−^ mice with the CD80/CD86–CD28 pathway deficiency [51]. In line with this, another research group reported that adoptive transfer of CD4^+^CD25^+^ Tregs attenuated the development of atherosclerosis in Treg-competent *Apoe*^−/−^ mice [52]. These findings provided the novel concept that CD4^+^CD25^+^ Tregs protect against atherosclerosis in mice under hypercholesterolemia. A recent study investigated the role of another costimulatory pathway, CD27–CD70, in atherosclerosis by inducing bone marrow-derived and systemic CD27 deficiency in *Apoe*^−/−^ mice, and showed that CD27 deficiency exacerbated early stages of atherosclerosis, along with reduced Treg numbers in various lymphoid organs and the aorta [53]. Interestingly, this study also demonstrated that adoptive transfer of wild-type CD4^+^CD25^+^ Tregs expressing folate receptor 4 into CD27-deficient *Apoe*^−/−^ mice reversed the phenotype of atherosclerosis, indicating a causative role of decreased Treg frequency in CD27-deficiency-dependent atherosclerosis progression [53].

An important issue for the definition of Tregs is that CD25-expressing CD4^+^ T cell population may contain activated conventional T cells. Firm experimental evidence supports that Foxp3 is the most reliable molecular marker for Tregs in mice [48]. To investigate the precise role of Tregs in atherosclerosis, Klingenberg et al. utilized the DEREG (depletion of regulatory T cells) mice, in which Foxp3^+^ Tregs faithfully express a diphtheria toxin receptor and can be specifically depleted by diphtheria toxin administration [54]. Treg deficiency by transplanting DEREG bone marrow into lethally irradiated *Ldlr*^−/−^ mice accelerated atherosclerosis development without affecting aortic inflammatory responses [55]. This pro-atherogenic effect caused by Treg depletion was associated with markedly elevated plasma cholesterol levels in the very low-density lipoprotein and chylomicron remnant fractions [55]. This study identified for the first time the role of Foxp3^+^ Tregs in atherosclerosis and demonstrated a novel suppressive mechanism that modulates lipid metabolism other than the well-recognized inflammation regulation.

### 3.2. Possible Protective Role of Tregs in Human Atherosclerosis

In addition to the strong experimental evidence for atheroprotective actions of Tregs, accumulating evidence highlights their importance in human atherosclerotic disease. Low numbers of FOXP3^+^ Tregs were detected in all the progression stages of human atherosclerotic plaques [56]. Single-cell RNA sequencing of a broad cohort of human carotid plaques identified a small sized cluster, showing Tregs detected by the expression of *FOXP3*, *CD25*, and *CTLA4* [57]. Although the number and phenotype of Tregs in human atherosclerotic lesions have not been extensively explored, there are a number of studies that examined the correlation between circulating Treg levels and coronary artery disease (CAD) [58,59,60,61,62], showing reduced peripheral Treg numbers in ACS patients compared with healthy controls or stable angina patients [58,61]. A recent large and long follow-up study showed an association between low levels of baseline CD4^+^FOXP3^+^ Tregs and an increased risk of acute coronary events, but not stroke [63]. The CD4^+^CD25^+^CD127^low^ Treg counts in coronary thrombi were significantly increased compared with peripheral blood in patients with ST elevation or non-ST elevation ACS, indicating that peripheral Tregs might decrease due to their accumulation in ACS lesions in the acute phase of MI [64]. Together, these results suggest that decreased numbers of Tregs may be responsible for the pathogenic inflammation in ACS.

Several markers, such as CD25, CD127, and FOXP3 have been used to define Tregs in previous clinical studies. The strategy of staining the FOXP3 molecule can discriminate Tregs from Teffs more precisely than by the combination of CD25 and CD127 molecules. However, as opposed to murine CD4^+^Foxp3^+^ T cells, FOXP3 expression is induced in human CD4^+^FOXP3^−^ T cells upon TCR stimulation, which do not exhibit suppressive functions, and therefore such a population may contain some Teffs [8]. Miyara et al. proposed a new strategy to define human Tregs, demonstrating that Tregs are separated into two subsets (CD4^+^CD45RA^+^FOXP3^low^ resting Tregs and CD4^+^CD45RA^−^FOXP3^high^ activated Tregs) and that activated Teffs are defined as CD4^+^CD45RA^−^FOXP3^low^ T cells or CD4^+^CD45RA^−^FOXP3^−^ T cells [65]. The combination of CD45RA and FOXP3 staining of CD4^+^ T cells may identify Tregs in human peripheral blood more precisely than previous methods. Using this strategy to accurately define human Tregs, we reported that both resting Treg and activated Treg levels were decreased, whereas the CD4^+^CD45RA^−^FOXP3^−^ fraction of activated Teffs was increased in the peripheral blood of patients with stable angina pectoris and old MI compared with healthy controls [66]. These results imply that dysregulated Treg responses might promote atherosclerosis in humans, though it remains unclear whether the imbalance between proatherogenic Teffs and atheroprotective Tregs is a cause or result of CAD. Another important issue regarding the difference in FOXP3 expression between mice and humans is that there are several different isoforms for FOXP3, including FOXP3 lacking the region encoded by exon 2 (FOXP3Δ2). A recent clinical study revealed that not total FOXP3 levels but the proportion of FOXP3Δ2 expression was associated with symptomatic atherosclerotic disease, and that Treg activation led to the induction of FOXP3Δ2 isoform expression that plays an important role in the regulation of its effector functions [67]. Collectively, these reports highlight the possible role of Tregs in the protection against human atherosclerotic disease.

### 3.3. Protective Role of Tregs in AAA

AAA characterized by dilation of the abdominal aorta is a lethal aortic disease and associated with atherosclerosis. Importantly, there are no effective pharmacological therapies against this disease, and surgical treatment is performed if the risk of rupture is higher than that of the procedure. Therefore, it is important to elucidate the detailed mechanisms underlying AAA development and develop noninvasive therapies for this disease. Clinical and experimental evidence suggests that inflammation caused by accumulation of Teffs and macrophages in aneurysmal lesions contributes to the development of AAA, and that Tregs protect against AAA formation [68]. Genetic deficiency of CD4^+^CD25^+^ Tregs promotes the development and rupture of angiotensin II-induced AAA in normocholesterolemic mice [69]. Adoptive transfer of CD4^+^CD25^+^ Tregs prevents angiotensin II-induced AAA formation in atherosclerosis-prone *Apoe*^−/−^ mice [70]. Using hypercholesterolemic DEREG mice, we demonstrated that genetic depletion of CD4^+^Foxp3^+^ Tregs aggravates AAA formation and rupture in angiotensin II-infused *Apoe*^−/−^ mice by upregulating immunoinflammatory responses in the aneurysmal lesions, providing direct evidence for a protective role of CD4^+^Foxp3^+^ Tregs in the development of AAA [71]. In patients with AAA, decreased numbers of CD4^+^CD25^+^FOXP3^+^ Tregs were observed, and Foxp3 expression in peripheral CD4^+^CD25^+^ cells was decreased compared with healthy control subjects [72]. FOXP3 expression levels in human aneurysmal tissues are significantly lower than in normal thoracic aortic tissues [70]. These reports suggest that impaired immunoregulation by Tregs may be involved in the development of AAA, and that promotion of endogenous regulatory immune responses may represent an attractive therapeutic approach to AAA.

### 3.4. Mechanisms of Treg-Mediated Atheroprotection

A large number of studies in immunology fields have elucidated the mechanisms by which Tregs control pathogenic immune responses, leading to the development of effective approaches to prevent and treat inflammatory diseases based on the modulation of their functions. Tregs exert suppressive functions via multiple mechanisms, including cell contact-dependent suppression, secretion of immunosuppressive factors including IL-10, IL-35, and transforming growth factor (TGF)-β, intracellular molecule (granzyme, cyclic adenosine monophosphate, and indoleamine 2,3-dioxygenase)-dependent suppression, and IL-2 deprivation from responder T cells, which could operate in a synergistical or complementary manner [8]. Although it remains obscure which mechanism is dominant for the Treg-mediated regulation of pathogenic immune responses, core suppressive pathways may depend on the type and stage of disease, or subsets of Tregs.

Among cytokines secreted from T cells, IL-10 and TGF-β have potent anti-inflammatory actions and have attracted much attention as potent anti-atherosclerotic cytokines [73]. Regarding the suppressive actions of Tregs in atherosclerosis, IL-10 and TGF-β production might be involved in this process. In *Ldlr*^−/−^ mice fed a high-cholesterol diet, overexpression of IL-10 in T cells inhibited the development of advanced atherosclerotic lesions by shifting the Th1/Th2 balance toward Th2 phenotype [74]. Genetic deletion of TGF-β signaling in T cells led to a dramatic increase in atherosclerotic lesion development with an unstable plaque phenotype in *Apoe*^−/−^ mice [75]. As described above, our recent study demonstrated that overexpression of CTLA-4 in T cells inhibited atherosclerosis development in *Apoe*^−/−^ mice by downregulating the CD80 and CD86 expression on DCs and limiting the CD80/CD86–CD28-dependent activation of Teffs [34]. CTLA-4-dependent suppression of DC function by Tregs may also be involved in the reduction in atherosclerosis. However, it remains to be determined whether these suppressive mechanisms depend on Tregs or other subsets of T cells. Tregs contribute to the promotion of inflammation resolution by enhancing apoptotic cell clearance (efferocytosis) by macrophages, which could contribute to the prevention of atherosclerosis development [76]. The identification of dominant atheroprotective mechanisms mediated by Tregs would lead to the establishment of novel Treg-based therapies for atherosclerotic disease.

### 3.5. Treg Immune Responses under Hypercholesterolemia

Several experimental reports have shown the close link between hypercholesterolemia and Treg number and function (Table 1). The proportion of Foxp3^+^ Tregs within the splenic CD4^+^ T cell population was markedly increased in *Ldlr*^−/−^ mice fed a cholesterol-rich diet, whereas their accumulation in atherosclerotic plaques was impaired in association with decreased expression of their surface molecules related to migratory function, which was prevented by reversal of hypercholesterolemia [77]. The in vitro suppressive function of Tregs was maintained under hypercholesterolemia, although their expression of various selectin ligands and binding capacity to aortic endothelium were decreased and apoptosis of intraplaque Tregs was promoted under such conditions [77]. Another experimental study in wild-type mice fed a cholesterol-rich diet demonstrated that diet-induced hypercholesterolemia rather increased an in vitro Treg suppressive activity [78]. In *Ldlr*^–/–^ mice fed a high-cholesterol diet exhibiting mild hypercholesterolemia, Treg differentiation was promoted in the liver [79]. However, under severe hypercholesterolemia, disrupted homeostasis in the liver promoted Th1 cell differentiation and CD11b^+^CD11c^+^ leukocyte accumulation, resulting in the abrogation of Treg responses and the promotion of atherosclerosis [79]. A recent study in *Ldlr*^−/−^ mice fed a high-cholesterol diet has shown that diet-induced hypercholesterolemia modulated the intracellular metabolism of Tregs and the expression of several molecules involved in their migration, which led to the conversion of Tregs to an effector-like migratory phenotype and the promotion of their migration towards atherosclerotic aortas [80]. These results imply that decreased Treg migratory capacity due to hypercholesterolemia may not be responsible for the dysfunction of their ability to control atherosclerosis. Thus, accumulating experimental evidence indicates that hypercholesterolemia affects the number, function, migratory capacity, and intracellular metabolism in Tregs. Further studies are needed to better understand these mechanisms.

It is supposed that Tregs in circulation enter the lymphoid tissues surrounding atherosclerotic arteries where they may be presented with some atherosclerosis-associated antigens by DCs, become activated, and migrate into atherosclerotic aortas to mitigate lesional inflammation and plaque development. This implies that the expansion of Tregs in atherosclerotic lesions could lead to reduced plaque inflammation and lesion development. The chemokine system plays an important role in the recruitment of T cells and monocytes to atherosclerotic lesions [81]. Several chemokine receptors are specifically expressed on each Th subset, whereas Treg-specific chemokine receptors have not been identified. An experimental report demonstrated that the expression of the chemokine CX3CL1 was selectively upregulated in the aorta of *Ldlr*^−/−^ mice fed a high-cholesterol diet compared with other lymphoid tissues, and that the adoptive transfer of its counterreceptor CX3CR1-transduced Tregs promoted their migration to the atherosclerotic lesions and reduced atherosclerosis development, although CX3CR1 is mainly expressed on Ly6C^+^ monocytes and its endogenous expression on Tregs is relatively low [82]. The role of chemokine–chemokine receptor interactions in Treg recruitment to atherosclerotic lesions remains largely unknown. Importantly, it remains unclear whether lesional Tregs suppress inflammatory reactions responsible for atherosclerotic lesion development, and further extensive experiments are required to identify the exact role of lesional Tregs in atherosclerosis.

### 3.6. Stability, Plasticity, and Antigen-Specificity of Tregs in Atherosclerosis

Tregs may stably exert suppressive activities for a long time under physiological conditions, while they have been reported to lose inhibitory functions (instability) and show an effector-like phenotype (plasticity) under inflammatory conditions (Table 1) [83]. Similar phenomena are observed under the conditions of prolonged hypercholesterolemia [84]. In *Apoe*^−/−^ mice, CD4^+^Foxp3^+^ Tregs could differentiate into proinflammatory Th1-like cells in the aorta and secondary lymphoid tissues and become dysfunctional, leading to the exacerbation of arterial inflammation and lesion development [85]. CD4^+^Foxp3^+^CCR5^+^CD25^−^ Tregs found exclusively in the aorta and draining lymph nodes of *Apoe*^−/−^ mice fed a high-cholesterol diet for a long period exhibit impaired suppressive activities and exacerbate atherosclerosis, although it remains unclear whether this cell population is derived from bona fide Tregs [86].

As the disruption of immunologic tolerance against modified lipoproteins may be responsible for the pathogenesis of atherosclerosis, it could be considered as an autoimmune disease. T cells including Tregs respond to some antigens derived from components of LDL particles [7]. MHC class II-deficient *Apoe*^−/−^ mice had greater atherosclerotic lesions and reduced numbers of Tregs, implying that MHC class II-mediated generation and activation of antigen-specific Tregs would have a protective role in the development of atherosclerosis [87]. However, due to technical limitations, the role of antigen-specific T cells in atherosclerosis has not been elucidated in most experimental or clinical studies. A recent study using newly designed tetramers to detect human T cells specific for ApoB-derived peptides ApoB_3036–3050_, possible atherosclerosis-related antigens, has shown that healthy subjects have ApoB-specific Foxp3^+^ Tregs, and that these Tregs coexpress other CD4 lineage transcription factors, such as ROR-γt, in patients with subclinical cardiovascular disease [88]. Another study by the same group using a tetramer to detect ApoB_978–993_-specific T cells has revealed that ApoB-specific T cells in *Apoe*^−/−^ mice convert from mixed Th17/Treg cells with a regulatory anti-inflammatory transcriptome into proinflammatory Th1/Th17-like cells that secrete inflammatory cytokines, and that these ApoB-specific CD4^+^ T cells with a predominant Th1/Th17 phenotype were detected in the blood of patients with CAD [89]. Many questions remain to be answered regarding the role of stability, plasticity, and antigen specificity of Tregs in atherosclerosis [7]. Further studies should address these issues to translate findings obtained by experimental studies to the clinical settings.

## 4. Therapeutic Approaches for Atherosclerotic Disease by Promoting Regulatory Immune Responses

**Figure 2:**
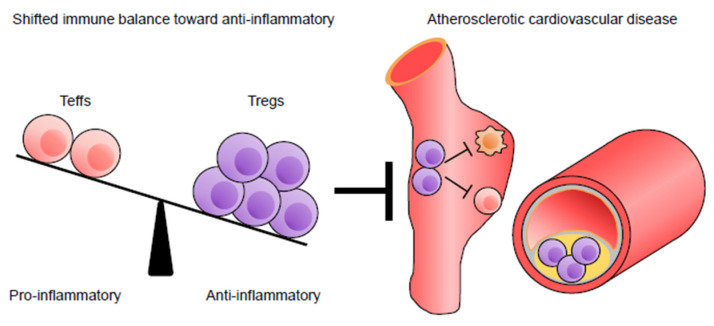
The balance between proatherogenic effector T cells (Teffs) and atheroprotective regulatory T cells (Tregs) is critical for the control of atherosclerosis.

The balance between Teffs and Tregs is critical for the control of atherosclerotic disease. Based on recent experimental and clinical evidence, we expect that improving this balance, by enhancing Treg responses or dampening Teff responses, could be an effective therapy for atherosclerotic disease [9,10] (Figure 2). A large number of experimental studies have shown that the activation and expansion of antigen-specific or non-specific Tregs protect against atherosclerosis by suppressing pathogenic immune responses (Table 2). This may be supported by the fact that upon antigen presentation, Tregs also exert suppressive functions in an antigen-non-specific manner [48].

### 4.1. Vaccination Strategies

Experimental studies in atherosclerosis-prone mice have demonstrated that immunization with several atherosclerosis-related antigens, native LDL, oxidized LDL, or ApoB-derived peptides, induces antigen-specific Tregs and attenuates atherosclerotic lesion development [16]. The induction of antigen-specific immunological tolerance could be an attractive therapeutic strategy for atherosclerosis, because this approach can dampen pathogenic immune responses against atherosclerosis-related antigens and avoid general immunosuppression. The identification of atherosclerosis-related antigens and the development of effective immunization methods would advance the field in designing a novel approach to prevent human atherosclerosis. For more in-depth information on vaccination strategies in atherosclerosis, the reader is referred to a recent review [16].

### 4.2. Modulation of DC Functions

The interaction between Tregs and some DC subsets play a protective role in atherosclerosis [9]. DC maturation mediated by MyD88, a key Toll-like receptor adaptor, is atheroprotective through controlling Treg responses in Western-type diet-fed *Ldlr*^−/−^ mice [90]. Flt3 signaling-dependent CD11c^hi^MHC-II^hi^CD11b^−^CD103^+^ DCs in aortic tissue promote accumulation of CD4^+^Foxp3^+^ Tregs and protect against atherosclerosis in *Ldlr*^−/−^ mice fed a high-fat diet [91]. Immature tolerogenic DCs, characterized by low expression levels of MHC class II molecule and costimulatory molecules, expand CD4^+^Foxp3^+^ Tregs and inhibit Teff responses, contributing to the maintenance of immune tolerance and regulation of atherosclerosis [92]. Adoptive transfer of ApoB100-pulsed tolerogenic DCs prevented atherosclerosis development in hypercholesterolemic mice by diminishing pathogenic T cell responses to ApoB100 and promoting antigen-specific CD4^+^Foxp3^+^ Treg responses [93]. Our study showed that oral administration of the active form of vitamin D_3_ (calcitriol) attenuated atherosclerosis development in *Apoe*^−/−^ mice by expanding tolerogenic DCs and CD4^+^Foxp3^+^ Tregs systemically and locally in atherosclerotic lesions, which might interact with each other and efficiently regulate aortic inflammatory responses associated with atherosclerosis [94]. This study provided direct evidence for a possible anti-atherogenic role of vitamin D reported in an epidemiological study showing the association between vitamin D deficiency and increased cardiovascular events and mortality [95]. Collectively, strategies to expand and activate several types of atheroprotective DCs could be possible therapies for the prevention of atherosclerosis [92].

### 4.3. Modulation of Intestinal Immunity

As described above, Foxp3^+^ Tregs (known as pTregs) differentiate from naïve T cells not only in the thymus but also in GALT, where multiple stimuli such as commensal microbiota and food antigens could effectively promote their induction [49,96]. T regulatory type 1 (Tr1) or T helper type 3 (Th3) cells that do not express Foxp3 are induced in GALT and produce immunomodulatory cytokines IL-10 or TGF-β, respectively [96]. The development of atherosclerosis was attenuated by in vivo induction of Tr1 cells in atherosclerosis-prone mice, which was associated with increased production of IL-10 [97,98]. Experimental evidence indicates that induction of mucosal (oral or nasal) tolerance may be an effective way to prevent and treat various autoimmune diseases, including atherosclerosis, and has attracted much interest, although clinical trials to examine the preventive effect of this approach on some autoimmune diseases were unsuccessful [96]. One of the critical mechanisms for mucosal tolerance induction is supposed to be induction of several types of self-antigen-specific Tregs described above [96]. Oral tolerance induction to oxidized LDL [98] or heat shock protein 60 [99] reduced the development of atherosclerosis in *Ldlr*^−/−^ mice, in association with increased proportion of CD4^+^CD25^+^Foxp3^+^ Tregs in several lymphoid organs and promoted the production of TGF-β or IL-10 in mesenteric lymph nodes upon each antigen stimulation, respectively. However, antigen-specificity of expanded Tregs was not clearly determined in these studies.

Gut microbiota are highly associated with the intestinal immunity and systemic metabolic disorders. Recent experimental and clinical studies have provided evidence for an association between gut microbiota composition and development of CVD [100]. By performing 16S ribosomal RNA gene sequencing in fecal samples from CAD patients and mechanistic studies with *Apoe*^−/−^ mice, we have recently demonstrated that the relative abundance of *Bacteroides vulgatus* and *Bacteroides dorei* was lower in patients with CAD compared with controls, and that this microbiota protects against atherosclerosis by ameliorating endotoxemia and systemic inflammation [101]. CD4^+^Foxp3^+^ Tregs were expanded in the colon lamina propria by oral administration of mouse [102] and human [103] *Clostridium* species, the spore-forming component of indigenous intestinal microbiota, and this Treg induction resulted in attenuation of experimental colitis. As the mechanisms for induction of colonic Tregs, butyrate, one of commensal bacteria-derived short-chain fatty acids, was shown to regulate the differentiation of Tregs and ameliorate the development of experimental colitis [104]. Although no prior reports investigated the role of *Clostridium* species or microbial-derived butyrate in atherosclerosis, a recent experimental study using hypertensive mice with or without atherosclerosis has reported that treatment with another short-chain fatty acid propionate prevents cardiac damage and atherosclerosis by regulating inflammatory responses, which was mainly dependent on Tregs [105]. Collectively, recent experimental and clinical data suggest that some specific gut microbiota and microbial-derived metabolites may modulate atherosclerosis. Further investigation will contribute to the development of novel therapies to prevent atherosclerotic disease through modulation of intestinal immune system.

### 4.4. Treatment with Antibodies and Cytokines

Intravenously administered anti-CD3 monoclonal antibodies, effective in treating autoimmune diabetes in mice [106] and humans [107], also improve atherosclerosis in mice [108] by regulating Teff immune responses and expanding CD4^+^CD25^+^ Tregs. Oral or nasal anti-CD3 antibody administration can induce CD4^+^LAP (latency-associated peptide)^+^ Tregs that suppress experimental autoimmune diseases in a TGF-β-dependent manner [109,110]. We orally treated *Apoe*^−/−^ mice with anti-CD3 antibodies and observed significantly reduced plaque formation and inflammation, associated with the expansion of CD4^+^LAP^+^ Tregs and CD4^+^Foxp3^+^ Tregs in mesenteric lymph nodes [111]. As a mechanism of this suppression, we speculate that expanded Tregs may move to other lymphoid organs or atherosclerotic lesions and suppress proatherogenic immune responses. We propose the novel idea that oral immune modulation could serve as an attractive therapeutic approach to atherosclerotic disease.

CD4^+^Foxp3^+^ Tregs highly express CD25, a component of the high-affinity IL-2 receptor, and vigorously proliferate in the presence of IL-2. Recombinant mouse IL-2/anti-IL-2 monoclonal antibody complexes (IL-2 complexes) efficiently and selectively expand CD4^+^CD25^+^Foxp3^+^ Tregs and attenuate atherosclerosis in *Ldlr*^−/−^ [112] or *Apoe*^−/−^ mice [113]. Induction of CD4^+^Foxp3^+^ Tregs by IL-2 complex treatment also provides protection against angiotensin II-induced AAA formation in *Apoe*^−/−^ mice [71]. These reports suggest that IL-2 complex treatment could be a possible strategy to prevent both atherosclerosis and AAA. Considering that suppressive functions of Tregs can be impaired under inflammatory conditions [114], we speculate that both Treg expansion and inhibition of Teff responses could be necessary to potently suppress atherosclerosis. In line with this idea, we found that the combination treatment with anti-CD3 antibodies and IL-2 complexes was effective in expanding Treg with activated phenotype and preventing atherosclerosis development in *Apoe*^−/−^ mice [115].

Interestingly, a recent observational cohort study in patients with chronic graft-versus-host disease demonstrated that low-dose IL-2 was safely administered in these patients, which was associated with marked and sustained expansion of Tregs and improvement of the disease manifestations in a substantial proportion of treated patients [116]. Another clinical study in patients with vasculitis induced by the hepatitis C virus showed that the administration of low-dose IL-2 increased the percentage of Tregs without adverse effects in all subjects and led to the improvement of vasculitis in most patients [117]. Based on the data obtained from experimental and clinical studies described above, a randomized, double-blind, placebo-controlled, phase I/II clinical trial in patients with stable ischemic heart disease and ACS (LILACS), has begun to assess the safety and efficacy of low-dose IL-2 in patients with CAD [118,119]. It is expected that interesting results of this trial will be reported in the future.

### 4.5. Ultraviolet B (UVB)-Based Phototherapy

It is historically clear that sunlight exposure is indispensable for the maintenance of our health. UV is categorized into UVA (320–400 nm), UVB (280–320 nm), and UVC (100–280 nm). As UVC is blocked by the atmosphere and the ozone layer, we receive UVA and UVB wavelengths from natural sunlight in daily life. UVB irradiation is known to have beneficial effects on the immune system due to its anti-inflammatory and immunosuppressive actions, although chronic excessive UVB exposure could cause skin cancer or infectious diseases [120]. Based on this, UVB-based phototherapy is an established treatment for various human skin diseases without severe adverse effects [121].

Our recent work revealed that broad-band UVB (a continuous spectrum from 280 to 320 nm with a peak around 313 nm) exposure attenuates the development and progression of atherosclerosis in *Apoe*^−/−^ mice, associated with a systemic increase in CD4^+^Foxp3^+^ Tregs with higher suppressive capacity and a decrease in proinflammatory IFN-γ production from T cells [122]. Langerhans cells (LCs), epidermal Langerin^+^ DCs having an important role in presenting antigens to T cells, are reported to regulate immunoinflammatory reactions following their activation by various environmental stimuli, such as UVB [123,124]. Using LC-depleted mice on an *Apoe*^−/−^ background, we clearly showed that LCs play an indispensable role in the systemic expansion of Tregs and attenuation of atherosclerosis development and progression, suggesting the skin immune system as a novel therapeutic target for atherosclerosis. We also investigated the effect of UVB irradiation on the development of atherosclerosis-related vascular disease, such as AAA, and found that UVB irradiation attenuated the development of angiotensin II-induced AAA under hypercholesterolemia and prevented death due to its rupture [125]. Further analysis using hypercholesterolemic DEREG mice revealed that genetic depletion of CD4^+^Foxp3^+^ Tregs abrogated the protective effect of UVB treatment, indicating an indispensable role of Tregs for UVB-mediated prevention of AAA formation.

Clinical studies in patients with skin autoimmune disease demonstrated that bath-psoralen UVA or narrow-band UVB (a narrow peak around 311 nm) therapy expanded CD4^+^CD25^+^Foxp3^+^ Tregs in the periphery and improved disease state [126,127]. The efficacy of UVB therapy may vary depending on its wavelengths, although the detailed mechanisms of UVB-mediated protective effects on skin diseases remain obscure. Within the UVB spectrum, narrow-band UVB is the most effective wavelength for treating psoriasis [121]. Likewise, there might be some specific UVB wavelengths that are effective in preventing atherosclerosis, although we have no information about this so far. If the issues regarding the efficacy and safety of UVB therapy are overcome, UVB phototherapy could be an attractive immunomodulatory strategy for preventing atherosclerotic CVD.

### 4.6. Approaches to Regress Atherosclerosis

A great number of human and animal studies have revealed mechanisms of atherosclerotic plaque development, whereas mechanisms that reverse the disease remain obscure. In a clinical study, very intensive statin therapy lowered LDL cholesterol levels and increased high-density lipoprotein cholesterol levels, resulting in atherosclerosis regression, indicating the importance of lipid control for regressing established plaques [128]. In addition, experimental studies using several mouse models of atherosclerosis regression have highlighted the involvement of immunoinflammatory reactions in the process of atherosclerosis regression [129,130,131], although the precise mechanisms underlying this process have not been completely elucidated. Using a new mouse model of atherosclerosis regression in *Ldlr*^−/−^, we found that Eicosapentaenoic acid induced atherosclerosis regression by modulating DC functions and reducing CD4^+^ T cell numbers without increasing CD4^+^CD25^+^Foxp3^+^ Tregs [132], supporting the clinical benefits of this drug for the treatment of CVD in Japanese hypercholesterolemic patients [133]. Using the same mouse model, we also reported that intravenous injection of anti-CD3 antibodies induced rapid regression of established atherosclerotic lesions, associated with increased proportion of CD4^+^CD25^+^Foxp3^+^ Tregs in the regressing plaques as well as in the lymphoid organs [134]. This study for the first time suggested the possibility that the expansion of Tregs might contribute to regressing atherosclerotic plaques in mice. In line with this, a recent experimental study using various mouse models of atherosclerosis regression has demonstrated that CD4^+^CD25^+^Foxp3^+^ Tregs accumulated in the regressing plaques, and played an indispensable role for inflammation resolution in the artery wall by regulating macrophage- and T cell-mediated pro-inflammatory responses [135]. Collectively, these reports imply that in addition to intensive lipid-lowering therapies, the promotion of Treg immune responses may represent an effective therapeutic approach for atherosclerosis regression.

## 5. Conclusions

It has now become evident that arterial inflammation caused by immune dysregulation critically contributes to the development of atherosclerotic CVD. Recent clinical studies have confirmed that an anti-inflammatory therapy could be a possible strategy to prevent atherosclerotic CVD in patients with past cardiovascular disease history [4,5,6]. Although anti-inflammatory pharmacological therapies seem to be safe and effective in treating experimental atherosclerosis in mice, general immunosuppression caused by such therapies would cause adverse immune responses in humans [3]. Moreover, it will take much cost to prescribe expensive drugs for a long time. Therefore, other strategies to dampen inflammatory responses in atherosclerosis should be considered. 

Notably, firm evidence indicating the involvement of the adaptive immunity including Teff and Treg immune responses in atherosclerosis has accumulated [7]. Pharmacological approaches, vaccination strategies, and cell transfer of Tregs have been shown to reduce atherosclerosis development in mice [9,10]. Strategies to prevent atherosclerosis through boosting regulatory immune responses seem to be attractive and could be clinically applied for the treatment of atherosclerotic disease. However, despite accumulated knowledge about the role of protective Tregs in experimental atherosclerosis, we still lack direct clinical evidence to support this idea. To translate a large number of important findings obtained by animal experiments to clinical settings, extensive clinical studies will be required.

## Figures and Tables

**Table 1:** Role of Tregs in atherosclerotic disease.

**Possible Protective Role of Tregs in Atherosclerosis**		
	**Description**	**References**
Mouse	Genetic deficiency of the CD80/CD86–CD28 signaling decreases CD4^+^CD25^+^ Tregs in lymphoid tissues and exacerbates atherosclerosis in *Apoe^−/−^* or *Ldlr^−/−^* mice.	[51]
	Adoptive transfer of CD4^+^CD25^+^ Tregs attenuates the development of atherosclerosis in Treg-competent *Apoe^−/−^* mice.	[52]
	Genetic deletion of Foxp3^+^ Tregs accelerates atherosclerosis development in *Ldlr^−/−^* mice.	[55]
Human	Low numbers of FOXP3^+^ Tregs are detected in all the progression stages of atherosclerotic plaques.	[56,57]
	Peripheral Treg numbers are reduced in CAD patients	[58,61,66]
**Possible mechanisms of Treg-mediated atheroprotection**		
Cytokine secretion	Overexpression of IL-10 in T cells inhibits the development of atherosclerosis.	[74]
	Genetic deletion of TGF-β signaling in T cells dramatically accelerates atherosclerotic lesion development with unstable plaque phenotype.	[75]
Cell–cell contact	Overexpression of CTLA-4 in T cells inhibits atherosclerosis development by downregulating the CD80 and CD86 expression on DCs.	[34]
Efferocytosis	Tregs enhance apoptotic cell clearance by macrophages.	[76]
**Treg immune responses under hypercholesterolemia**		
Cell number	The proportion of splenic CD4^+^Foxp3^+^ Tregs is markedly increased.	[77]
	Treg differentiation is promoted in the liver under mild hypercholesterolemia.	[79]
Function	The expression of Treg surface molecules related to migratory function is decreased.	[77]
	Hypercholesterolemia increases in vitro Treg suppressive activity.	[78]
	Hypercholesterolemia modulates the intracellular metabolism of Tregs and promotes their migration towards atherosclerotic aortas.	[80]
	CD4^+^Foxp3^+^ Tregs differentiate into Th1-like cells in the aorta and secondary lymphoid tissues and become dysfunctional.	[85]

*Apoe*^−/−^, apolipoprotein E-deficient; CAD, coronary artery disease; CTLA-4, cytotoxic T-lymphocyte-associated antigen-4; DC, dendritic cell; Foxp3, forkhead box P3; IL, interleukin; *Ldlr*^−/−^, low-density lipoprotein receptor-deficient; TGF, transforming growth factor; Th1, T helper type 1; Treg, regulatory T cell.

**Table 2:** Strategies to prevent or treat atherosclerosis by promoting regulatory immune responses.

Strategies	Treatment	Immune Effects	References
Vaccination	Treatment with native LDL, oxidized LDL, or ApoB-derived peptides	Induction of antigen-specific Tregs	[16]
Modulation of DC functions	Transfer of ApoB100-plused tolerogenic DCs	Promoted antigen-specific CD4^+^Foxp3^+^ Treg responses and suppressed pathogenic T cell responses to ApoB100	[93]
	Oral administration of active form of vitaminD_3_ (calcitriol)	Increased tolerogenic DCs and CD4^+^Foxp3^+^ Tregs	[94]
Modulation of intestinal immunity	Oral tolerance induction to oxidized LDL or heat shock protein 60	Increased CD4^+^CD25^+^Foxp3^+^ Tregs and promoted production of TGF-β or IL-10 in mesenteric lymph nodes	[98,99]
	Oral administration of short-chain fatty acid propionate	Suppressed inflammatory responses mainly dependent on Tregs	[105]
Treatment with antibodies and cytokines	Intravenous administration of anti-CD3 monoclonal antibodies	Increased CD4^+^CD25^+^Foxp3^+^ Tregs and suppressed Teff immune responses	[108,134]
	Oral administration of anti-CD3 monoclonal antibodies	Increased CD4^+^LAP^+^ or CD4^+^Foxp3^+^ Tregs in mesenteric lymph nodes and suppressed Teff immune responses	[111]
	Treatment with recombinant mouse IL-2/anti-IL-2 monoclonal antibody complexes	Increased CD4^+^CD25^+^Foxp3^+^ Tregs and suppressed Teff immune responses	[112,113]
	Combination treatment with anti-CD3 monoclonal antibodies and IL-2 complexes	Increased CD4^+^Foxp3^+^ Tregs with activated phenotype	[115]
UVB-based phototherapy	UVB irradiation to the skin	Increased CD4^+^Foxp3^+^ Tregs and decreased IFN-γ production from T cells	[122]

ApoB, apolipoprotein B; DC, dendritic cell; Foxp3, forkhead box P3; IFN, interferon; IL, interleukin; LAP, latency-associated peptide; LDL, low-density lipoprotein; Teff, effector T cell; TGF, transforming growth factor; Treg, regulatory T cell; UVB, ultraviolet B.
